# Author Correction: The dynamics of plasmon-induced hot carrier creation in colloidal gold

**DOI:** 10.1038/s41467-025-58580-1

**Published:** 2025-04-03

**Authors:** Anna Wach, Robert Bericat-Vadell, Camila Bacellar, Claudio Cirelli, Philip J. M. Johnson, Rebeca G. Castillo, Vitor R. Silveira, Peter Broqvist, Jolla Kullgren, Alexey Maximenko, Tomasz Sobol, Ewa Partyka-Jankowska, Peter Nordlander, Naomi J. Halas, Jakub Szlachetko, Jacinto Sá

**Affiliations:** 1 SOLARIS National Synchrotron Radiation Centre, Jagiellonian University, Krakow, Poland; 2 Paul Scherrer Institut, Villigen PSI, Switzerland; 3 Institute of Physical Chemistry, Polish Academy of Sciences, Warsaw, Poland; 4 Department of Chemistry-Ångström, Physical Chemistry division, Uppsala University, Uppsala, Sweden; 5 Max Planck Institute for Chemical Energy Conversion, Mülheim an der Ruhr, Mülheim an der Ruhr, Germany; 6 Maxepartment of Chemistry-Ångström, Structural Chemistry division, Uppsala University, Uppsala, Sweden; 7 Department of Electrical and Computer Engineering, Rice University, Houston, TX USA; 8 Department of Physics and Astronomy, Rice University, Houston, TX USA; 9 Department of Chemistry, Rice University, Houston, TX USA

**Keywords:** Nanoparticles, Chemical physics, Photochemistry

Correction to: *Nature Communications* 10.1038/s41467-025-57657-1, published online 07 March 2025

In the version of the article initially published, the colours in the Fig. 2D “X-ray FEL” key were incorrect and have now been amended so that red represents “Excited State” and black represents “Steady State”. This correction has been made to the HTML and PDF versions of the article.

