# Supplementary material for: The dynamics of plasmon-induced hot carrier creation in colloidal gold
Source: Nat Commun. 2025 Mar 7;16:2274. doi: 10.1038/s41467-025-57657-1 (PMC11885627; doi:10.1038/s41467-025-57657-1)
Supplement: Supplementary file 1 — Supplementary information [file 41467_2025_57657_MOESM1_ESM.pdf]

Supplementary information

The Dynamics of Plasmon-Induced Hot Carrier Creation in Colloidal Gold

Anna Wach[1,2,3], Robert Bericat-Vadell[4], Camila Bacellar[2], Claudio Cirelli[2], Philip J. M. Johnson[2], Rebeca G. Castillo[5], Vitor R. Silveira[4], Peter Broqvist[6], Jolla Kullgren[6], Alexey Maximenko[1], Tomasz Sobol[1], Ewa Partyka-Jankowska[1], Peter Nordlander[7,8], Naomi J. Halas[7,8,9], Jakub Szlachetko[1*], Jacinto Sá[3,4*]

[1] SOLARIS National Synchrotron Radiation Centre, Jagiellonian University, Krakow, Poland

[2] Paul Scherrer Institut, CH-5232 Villigen PSI, Switzerland.

[3] Institute of Physical Chemistry, Polish Academy of Sciences, 01-224 Warsaw, Poland.

[4] Department of Chemistry-Ångström, Physical Chemistry division, Uppsala University, 751 20 Uppsala, Sweden.

[5] Max Planck Institute for Chemical Energy Conversion, Mülheim an der Ruhr D-45470, Germany.

[6] Department of Chemistry-Ångström, Structural Chemistry division, Uppsala University, 751 20 Uppsala, Sweden.

[7] Department of Electrical and Computer Engineering, Rice University, Houston, TX, USA.

[8] Department of Physics and Astronomy, Rice University, Houston, TX, USA.

[9] Department of Chemistry, Rice University, Houston, TX, USA.

*jakub.szlachetko@uj.edu.pl; jacinto.sa@kemi.uu.se

Materials and Methods:

Gold nanoparticles (Au NPs) preparation

The Au NPs were prepared using the Turkevich method, as outlined by Piella et al.[1] In a brief overview, a 50 mL solution of sodium citrate tribasic dihydrate (6.6 mM) in water was placed in a 100 mL round bottom flask and stirred at 70 °C in an oil bath. Subsequently, 0.1 mL of tannic acid (2.5 mM) was introduced to the reaction mixture. Finally, 1 mL of $HAuCl_4$ (25 mM) was promptly added. Within 5 minutes, the reaction mixture underwent a noticeable colour change from dark blue to wine, confirming the formation of the Au nanoparticles. The synthesised Au nanoparticles were then stored in a refrigerator.

Samples characterization

UV-Vis measurements:

The UV-Vis spectra were collected using a Cary 5000 UV-VIS-NIR spectrophotometer.

Dynamic Light Scattering (DLS) measurements:

The DLS data was collected in a Malvern Zetasizer nanoS instrument, and a total of 3 measurements comprised of 12 scans each time was done.

Transmission electron microscopy (TEM) measurements:

Bright field (BF) and high-resolution TEM images were recorded on a Transmission Electron Microscope Tecnai G2 operating at 200 kV containing an energy dispersive X-ray (EDX) microanalyzer and a High Angle Annular Dark Field Detector (HAADF). The sample for observation was prepared by drop-coating of Au NPs solution onto a 200 mesh copper grid. The images were then analyzed using ImageJ software.

Transient absorption spectroscopy (TAS)

A 40-fs pulsed laser with a 3 kHz repetition rate was generated through the Libra Ultrafast Amplifier System designed by Coherent. An optical parametric oscillator (TOPAS- prime, Light Conversion) generated the excitation beam. The signals were detected with a UV-NIR detector and a Newport MS260i spectrograph with interchangeable gratings. The fundamental laser (probe, 795 nm) passes through the delay stage (1-2 fs step size) and is focused in a CaF_2 optical window to generate visible light from 400 to 750 nm. The instrument response

function obtained for our system is ca. 95 fs. To regualate the intensity of the pump, a variable neutral density filter placed before the cell is used. The power of the pump is measured using a LM-2 Vis smiconductor power sensor by Coherent, and it is ensured that the measured power does not present a standard deviation above 2%. The pump diameter at the sample of approximately 370 µm ad for each measurement, at least 4 scans are performed and subsequently averaged to decrease the noise. The plasmonic electron-phonon lifetime ($\tau_{e\text{-}ph}$) was extracted by fitting the decay of the bleach at 500 nm. The kinetic traces were fitted using a sum of convoluted exponentials, following the methodology detailed in previous publication,[2] with the mathematical expression:

$$S(t) = e^{\left[-\left(\frac{t-t_0}{t_p}\right)^2\right]} * \sum A_i \, e^{\left(-\frac{t-t_0}{\tau_i}\right)} \qquad (eq.\ S1)$$

Where $t_p = \frac{IRF}{2ln2}$ and IRF is the width of the instrument response function (full width half maximum), t_0 is the time zero, A_i and τ_i are the amplitude and the decay times respectively, and * is the convolution operator.

Steady-state X-ray absorption near-edge structure (XANES):

The steady-state X-ray absorption spectra were collected at the ASTRA beamline of the SOLARIS National Synchrotron Radiation Center[3] in Krakow, Poland. The X-ray beam from the bending magnet (1.3 T) was monochromatised by a double crystal monochromator and focused to a 10 x 1 mm spot size on the sample. The L_3-edge XANES spectra of gold (Au L_3-edge 11919 eV) and platinum (Pt L_3-edge 11564 eV) metal foils were measured in transmission mode with the energy step size of 0.2 eV. Au NPs in aqueous solution were measured in a custom-made cell in a fluorescence mode using a one-element silicon drift detector (AXAS-M SDD, Ketek).

Standard XAS data reduction steps were performed with Athena software[4]. These include subtraction of pre- and post-edge backgrounds, determining the edge energy, and normalising the data set to an edge jump of 1.

Valence X-ray photoelectron spectroscopy (XPS):

For measurement, the synthesised gold nanoparticles were deposited on the fluorine-doped tin oxide (FTO) glass. Before sample preparation, the glass was cleaned by sonication in a detergent solution, Mili Q water and finally, isopropanol. Subsequently, the Au NPs suspension was mixed with 0.1 M HNO_3 in a 5 μL nitric acid ratio for each 100 μL Au NPs suspension. The cleaned FTO glass was then immersed in the solution overnight. Finally, the samples were dried in argon and annealed at 450°C for 30 min.

The valence band X-ray photoelectron spectra for the gold nanoparticles supported on the FTO glass were measured at the PHELIX beamline[5] of the SOLARIS synchrotron. The spectra were recorded with an electrostatic hemispherical analyser manufactured by SPECS GmbH, PHOIBOS 225, characterised by energy resolution better than 2 meV. XPS measurements were performed under UHV conditions and at room temperature, using monochromatic radiation at a photon energy of 1400 eV with an energy step of 50 meV. Sn *4d* peak was used to normalise the intensity of the spectra collected for Au NPs-FTO sample and the blank FTO. The valence region photoemission spectra were background subtracted with the range recorded for bare FTO.

SwissFEL experiments & data analysis:

The femtosecond X-ray absorption spectroscopy measurements were carried out at the Alvra endstation of the Swiss Free Electron Laser (SwissFEL, Paul Scherrer Institut, Switzerland). The experiment was performed in a PRIME chamber under a helium atmosphere (800 mbar pressure) to maximise X-ray transmission and minimise X-ray scattering noise. The aqueous solution of Au NPs (5 mM) was flown through a capillary to form a 100 μm diameter cylindrical liquid jet with a flow rate of 3-5 ml/min. The jet speed was selected to ensure the sample's renewal between consecutive laser pump/X-ray probe pulses. The liquid sample was pumped into the chamber by an HPLC pump, retrieved by a catcher system and then pumped out of the experimental chamber back into the sample reservoir by a peristaltic pump.

The sample was optically excited at 532 nm with a pulse duration of approximately 75 fs, generated at a 50 Hz repetition rate by an 800 nm Ti:Sapphire amplified laser system. The pump laser fluence (98 mJ/cm^2) was determined at the beginning of the experiment and chosen to maximise the excited-state fraction while minimising multiphoton absorption effects. The laser beam (focused into 60 x 60 μm^2 spot) and X-ray beam with a spot size of 20

x 20 μm^2, hit the sample in a nearly collinear geometry. The X-ray absorption data were recorded by scanning FEL electron beam energy and the Si(111) monochromator central energies across the Au L_3-edge (in the energy range 11890 – 11950 eV). The X-ray absorption data were recorded by an avalanche photodiode (APD detector) in total fluorescence yield (TFY) mode at approximately 90° from the incident X-ray beam. The fluorescence signal acquired over 5000 shots per energy point was normalised by the incoming X-ray pulse intensity (I0) on a pulse-to-pulse basis. The FEL was running at a repetition rate of 100 Hz, twice the laser pump, allowing for the measurement of consecutive laser-on and laser-off pulses to generate the transient difference signals.

The time scans (temporal traces) are an average of several individual scans collected over several thousand XFEL shots at 11916 eV (4.3 eV below Au E_F) and 11922 eV (1.7 eV above Au E_F). Following what has been published elsewhere,[6] the time traces were fitted with a monoexponential decay ($D_1(t)$) function described below:

$$D_1(t) = \frac{1}{2} * e^{-\frac{1}{t_1}\left(t-t_0-\sigma^2/t_1\right)} * \left[1 + IRF\left(\frac{t-t_0-\sigma^2/t_1}{\sqrt{2}\,\sigma}\right)\right] \qquad (eq.\ S2)$$

$$I(t) = a_1 * \ D_1(t) + C \qquad (eq.\ S3)$$

where $I(t)$ is the intensity of the pump-probe signal at a given time-delay t; t_0 is time-zero; σ is the full-width half maximum (FWHM) of the instrument response function (IRF), a_1 is the pre-exponential factor to the exponential decay time constant with $t_1$time constant and C is a constant offset.

The computer code for the temporal trace fitting was implemented in the Python language using a Jupyter notebook. The fitting process was done by the curve_fit function module from the SciPy Python package.[7] The module uses non-linear least squares to fit a function (user-defined) to data. Non-linear least squares analysis is used to fit a set of observations with a model that is non-linear with respect to the unknown parameters. The standard deviation errors on the fitting parameters were calculated based on the square-root procedure and a linear approximation to the model function around the optimum.

The transient XANES spectra (collected at time delays of 0, 100, 250, and 500 fs) were fitted with two Gaussian distribution peak functions to map signals below and above the Fermi

energy. Built-in Gaussian function in Origin Pro (2023b) was used to fit the peaks. The mean energy and width (3σ) distributions of charges (holes) were extracted from the area and FWHM of the Gaussian peak (below the E_F).

$$Gaussian\ funtion: \qquad y = y_0 + \frac{A}{\left(w * \left(\sqrt{\left(\frac{\pi}{4 * ln\,(2)}\right)}\right) * e^{\left(\frac{-4*ln(2)*(x-x_c)^2}{w^2}\right)}\right)} \qquad (eq.\ S4)$$

where A is the area, w is the full-width half maximum (FWHM), x_c is the peak center, and y_0 is the base.

The best fitting was decided based on mean-square error estimations, the standard method implemented in most software to fit ultrafast data.[8-10] The mean squared error of a procedure for estimating an unobserved quantity measures the average of the squares of the errors, that is, the average squared difference between the estimated values and the actual value. Mean squared error is a risk function, corresponding to the expected value of the squared error loss. The complete derivation and explanation of the method's strength are presented in several textbooks and can be found in the tutorial.[11]

Theoretical calculations of Au DOS:

The electronic structure calculations performed in this study were made using the self-consistent-charge density-functional based tight-binding method (SCC-DFTB)[12,13] as implemented in the DFTB+ software package.[14] In the calculations, we used the Slater-Koster tables generated in Ref. 4. This method has been shown to give band structures and electronic properties for different bulk phases and gold clusters (Au_2, Au_8 and Au_{20}) in excellent agreement with plane-wave density functional theory data.[15]

In the current work, we used SCC-DFTB to compute the electronic density of states of the Au bulk and for nanoparticles (NPs) of different sizes and shapes. The bulk Au was calculated using an fcc primitive unit cell sampled using a Monkhorst-Pack k-point grid of 9x9x9 using experimental lattice parameters. The nanoparticles were simulated in a large periodically repeated box tested only at the Gamma point. All structures were first geometry optimised until the maximum force on each atom was smaller than 5 meV/Å, keeping the supercell dimensions fixed.

Eleven nanoparticle sizes varying from 0.5nm to 3.3 nm in size (13 to 923 Au atoms) were investigated. The density of states (DOS) for these structures is shown in Figure S6. In the figure, it is seen that the DOS is size-dependent for the smaller sizes. However, for the larger ones, the electronic properties converge. Based on these calculations, we have chosen the 3.3 nm Au particle to represent larger Au NPs, which is used in the comparison to experimental data.

To further analyze the band occupations during the excitation process, we have performed electron-nuclear dynamics within the Ehrenfest ansatz as implemented in the DFTB+ code.[16] We used three differently sized Au NP's to investigate the size dependence. Starting from the equilibrium geometries for the icosahedrally shaped Au_{25}, $Au_{144,}$ and Au_{309} particles, we excited the particles using a shot laser pulse of 2 eV, explicitly allowing all atoms to move. 2 eV corresponds approximately to the energy at the start of the d-bands in the Au particles, as illustrated in Fig. S6. The short pulse will drive the electrons from occupied to unoccupied states, impulsively driving the Au nuclei to move. During the simulations, the occupation of each orbital was monitored and the resulting density of states of the holes-electron pairs around the Fermi level, as shown in Fig. S11.

Additional supporting data:

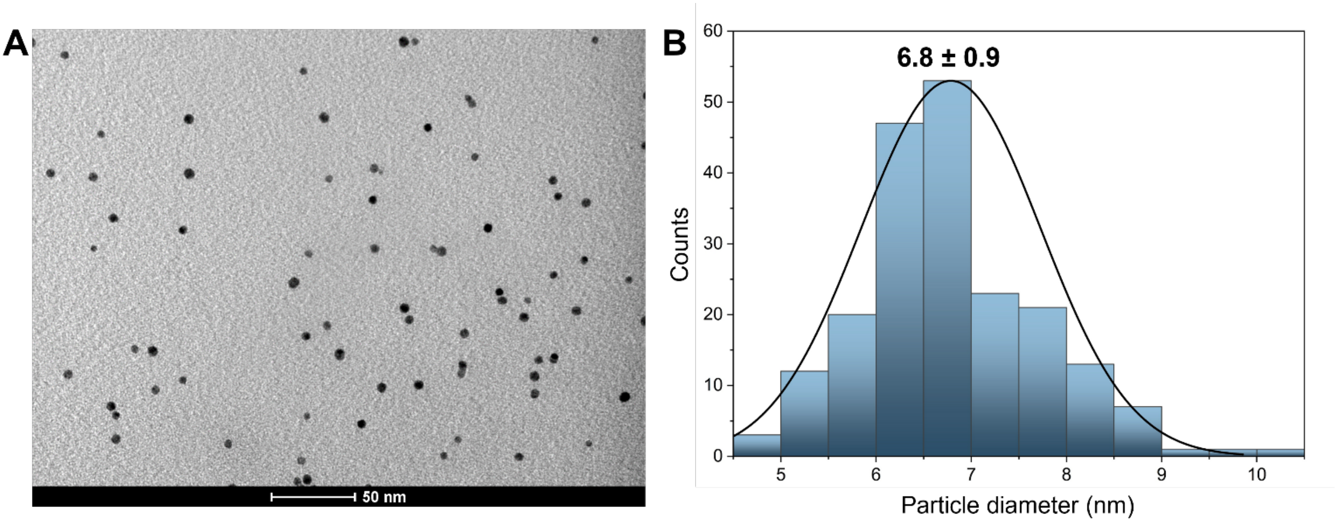

FIGURE S1: TEM analysis of Au NPs aquesous solution. A) TEM image with a scale bar; and (B) histogram depicting the particle size distribution.

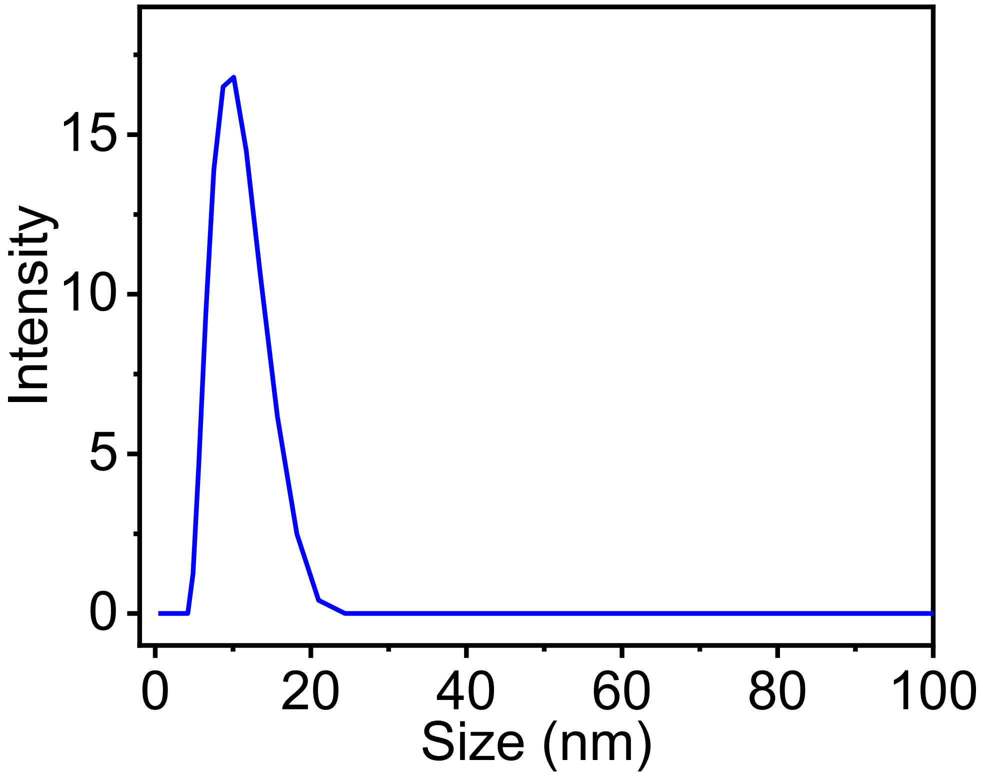

FIGURE S2: Dynamic light scattering (DLS) analysis of the used Au NPs in water.

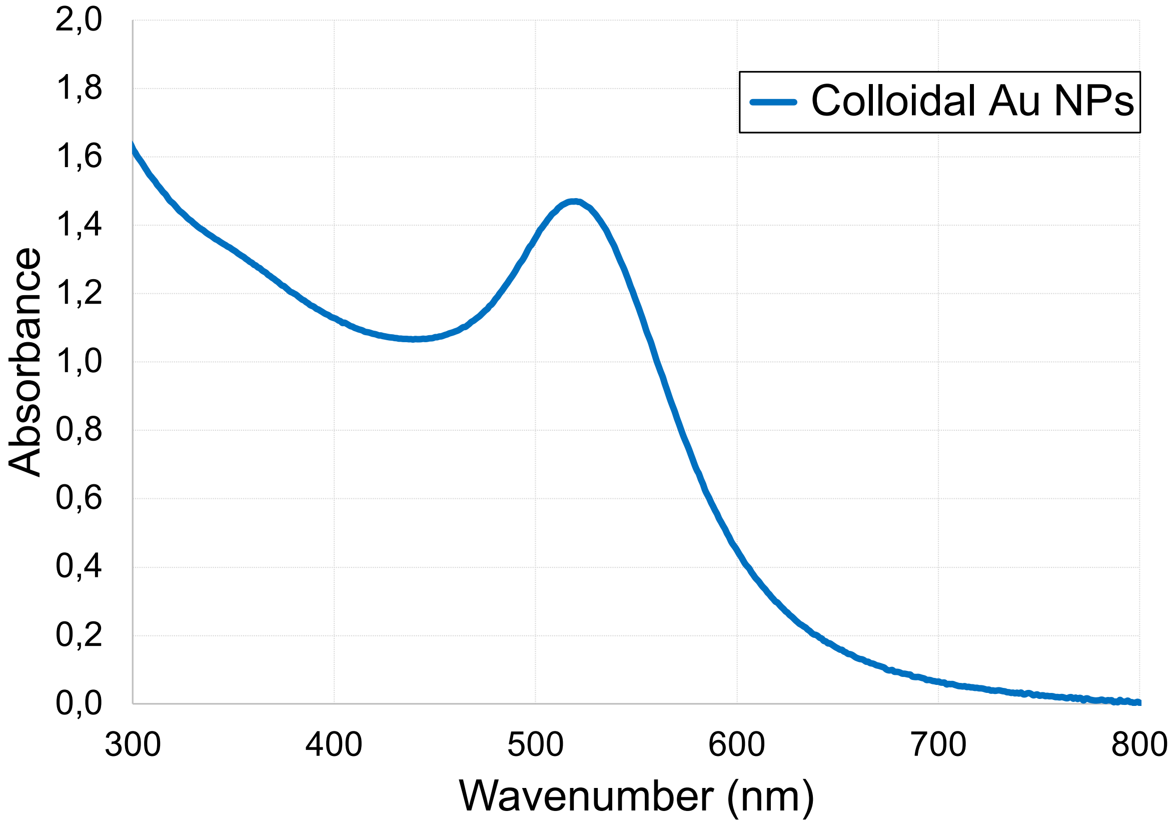

FIGURE S3: Optical absorption spectrum of colloidal Au NPs in water, measured without any additional processing. Please note that the UV-Vis spectrum relates to the colloidal solution after synthesis, whereas for the TR-XAS measurements, the sample was diluted fivefold.

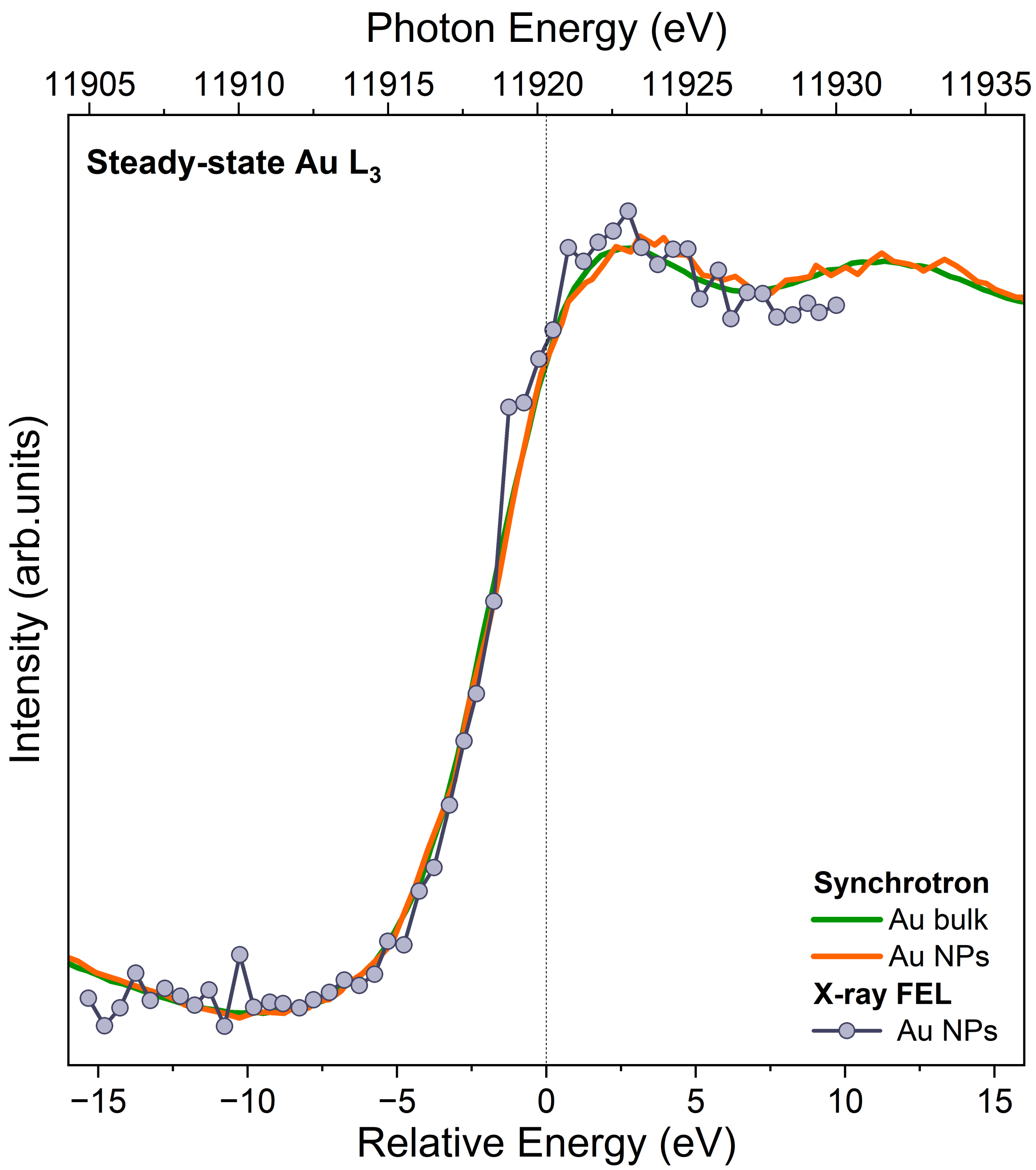

FIGURE S4: Steady-state Au L_3-edge X-ray absorption near edge structure spectra collected for Au foil and nanoparticles at synchrotron (Solaris, Poland) and X-ray free-electron laser (SwissFEL, Switzerland) facilities. The spectra are vertically shifted for clarity.The spectrum of Au NPs collected at SwissFEL was averaged over all scans measured without laser pulse (steady-state).

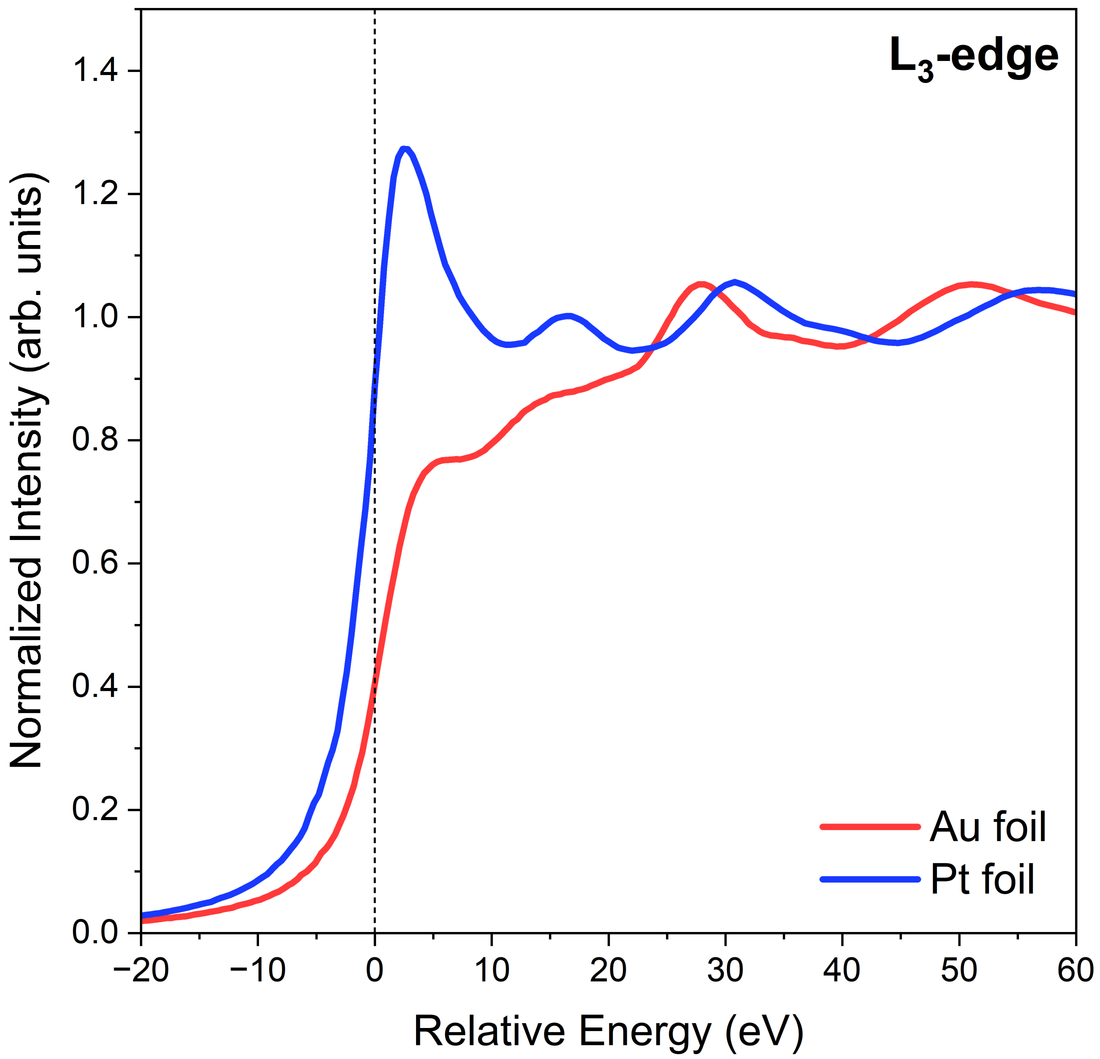

FIGURE S5: L_3-edge XANES of Au and Pt foils overplotted by normalizing the X-axis to the respective Fermi level energy. The inflection point in the edge (marked with a dashed line) was taken as the zero point in the energy scale. The foils were measured at the synchrotron (Solaris, Poland).

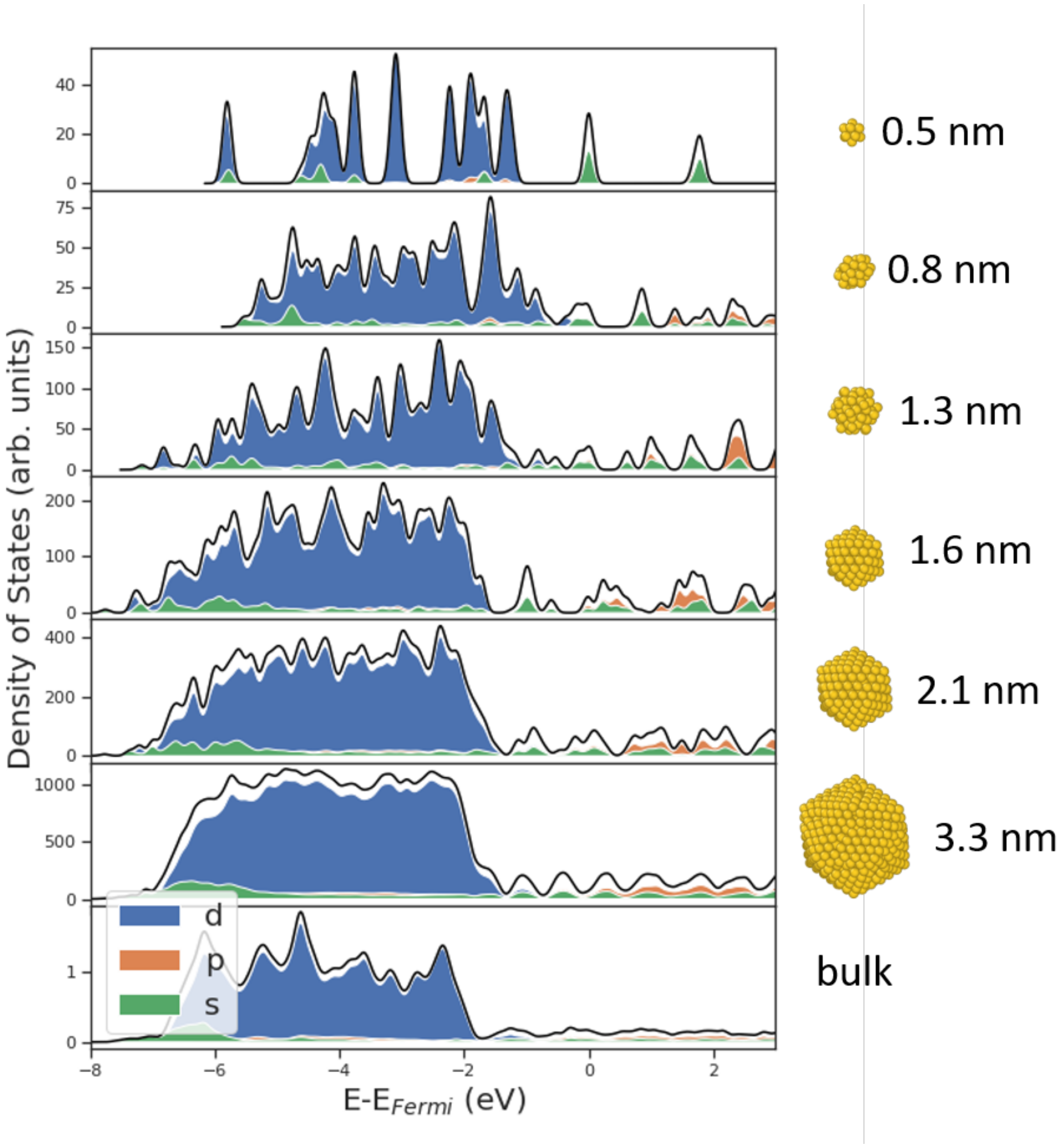

FIGURE S6: Density of states (DOS) for Au NPs of varying icosahedral size and bulk fcc Au.

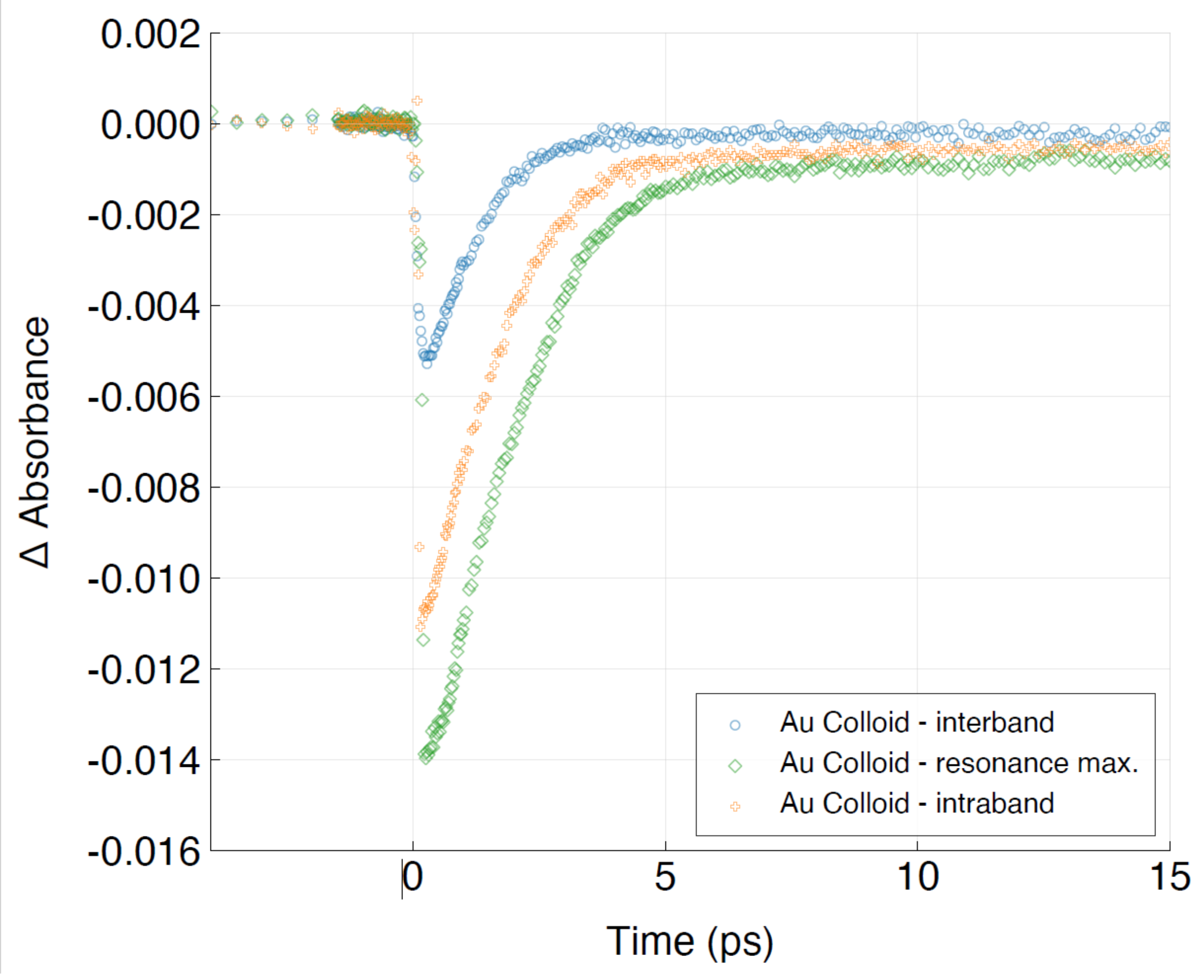

FIGURE S7: Kinetic traces extracted near the excitation wavelength, showing signal intensity after excitations at interband (below the LSPR peak at 450 nm), resonance maximum (at the LSPR peak maximum 520 nm), and intraband (above the LSPR peak at 532 nm).

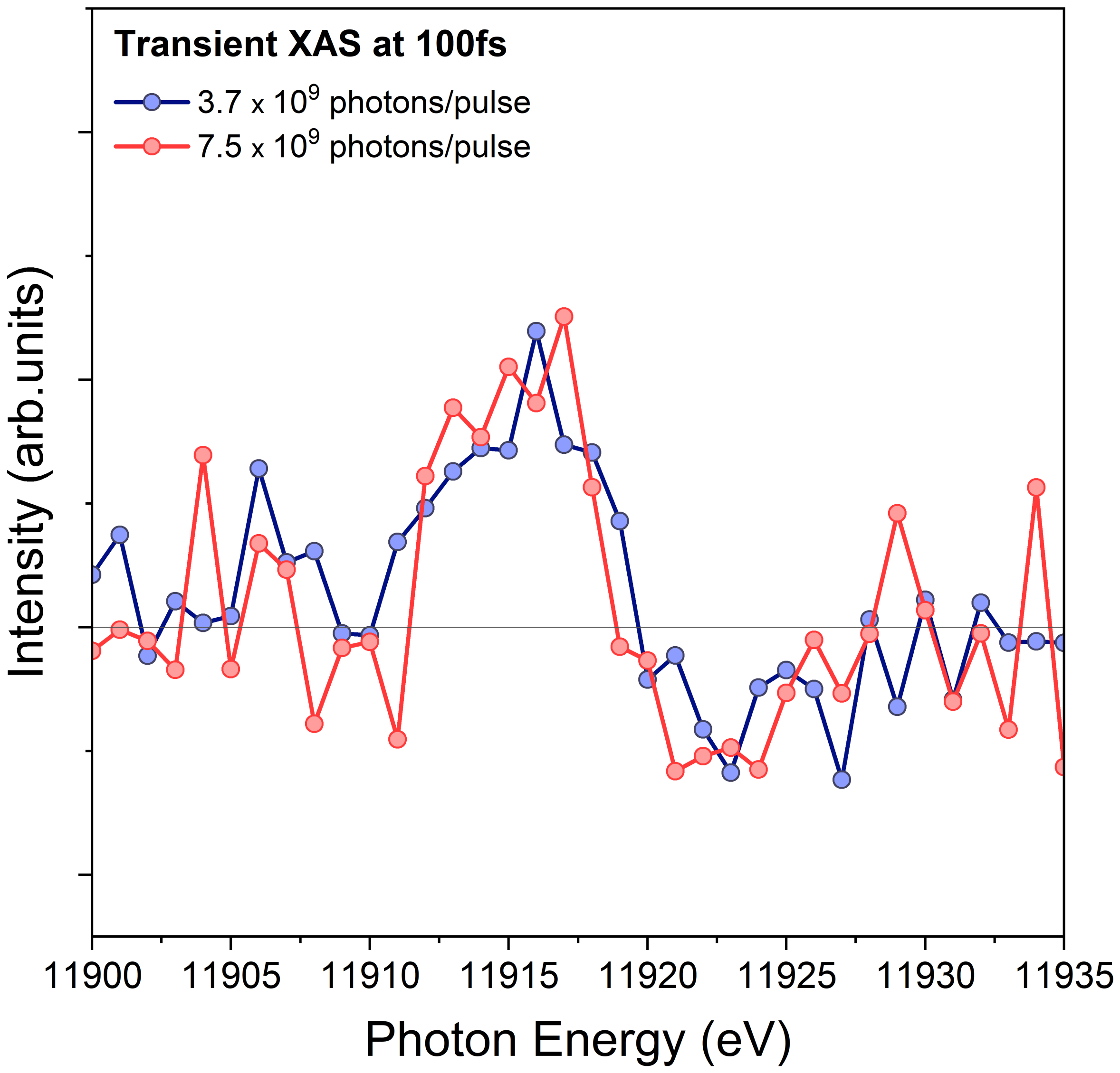

FIGURE S8: Comparison of transient XAS measured at 100 fs time delay and two X-ray fluxes.

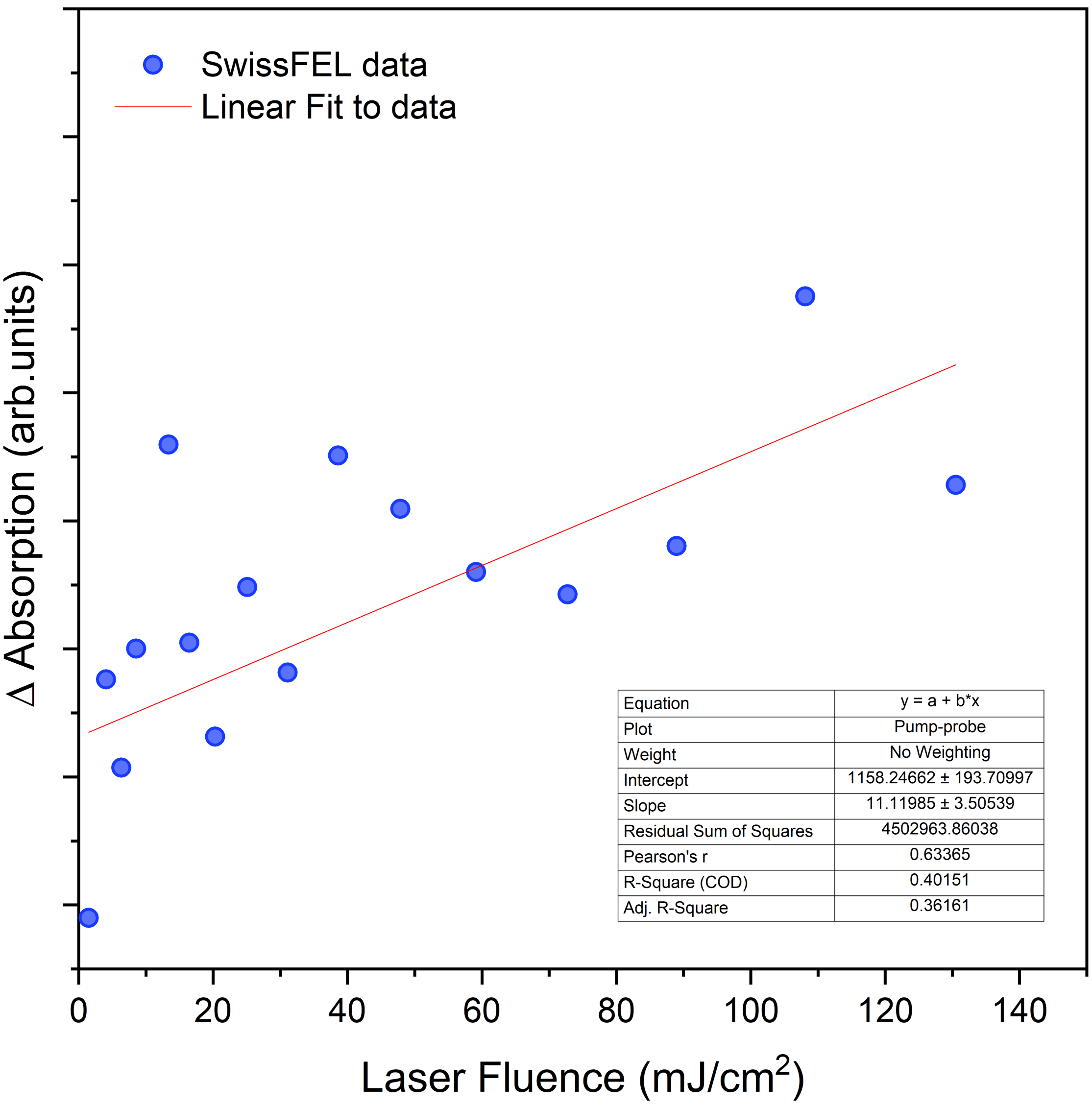

FIGURE S9: Fluence scan measured at SwissFEL with an incident energy of 11916 eV and a pump-probe delay of 100 fs. The scan shows a slight increase (slope intensity vs laser fluence 11.1 ± 3.5), indicating that the overall electron temperature remains relatively constant above a fluence of 5 mJ/cm².

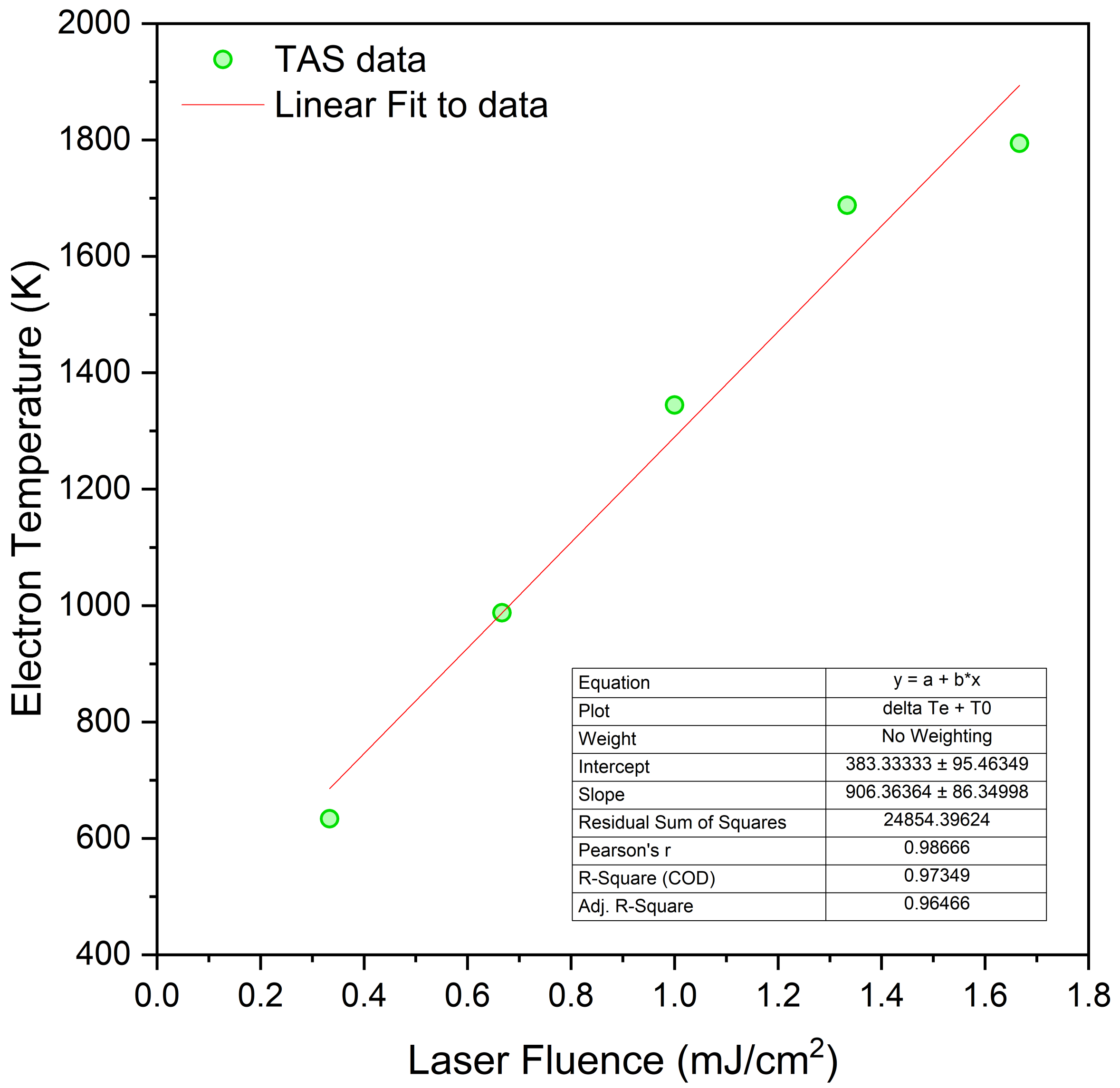

FIGURE S10: TAS laser fluence scan measured at a pump-probe delay of 100 fs, showing a significant increase in electron temperature with increasing laser fluence, with a slope of 383 ± 95.

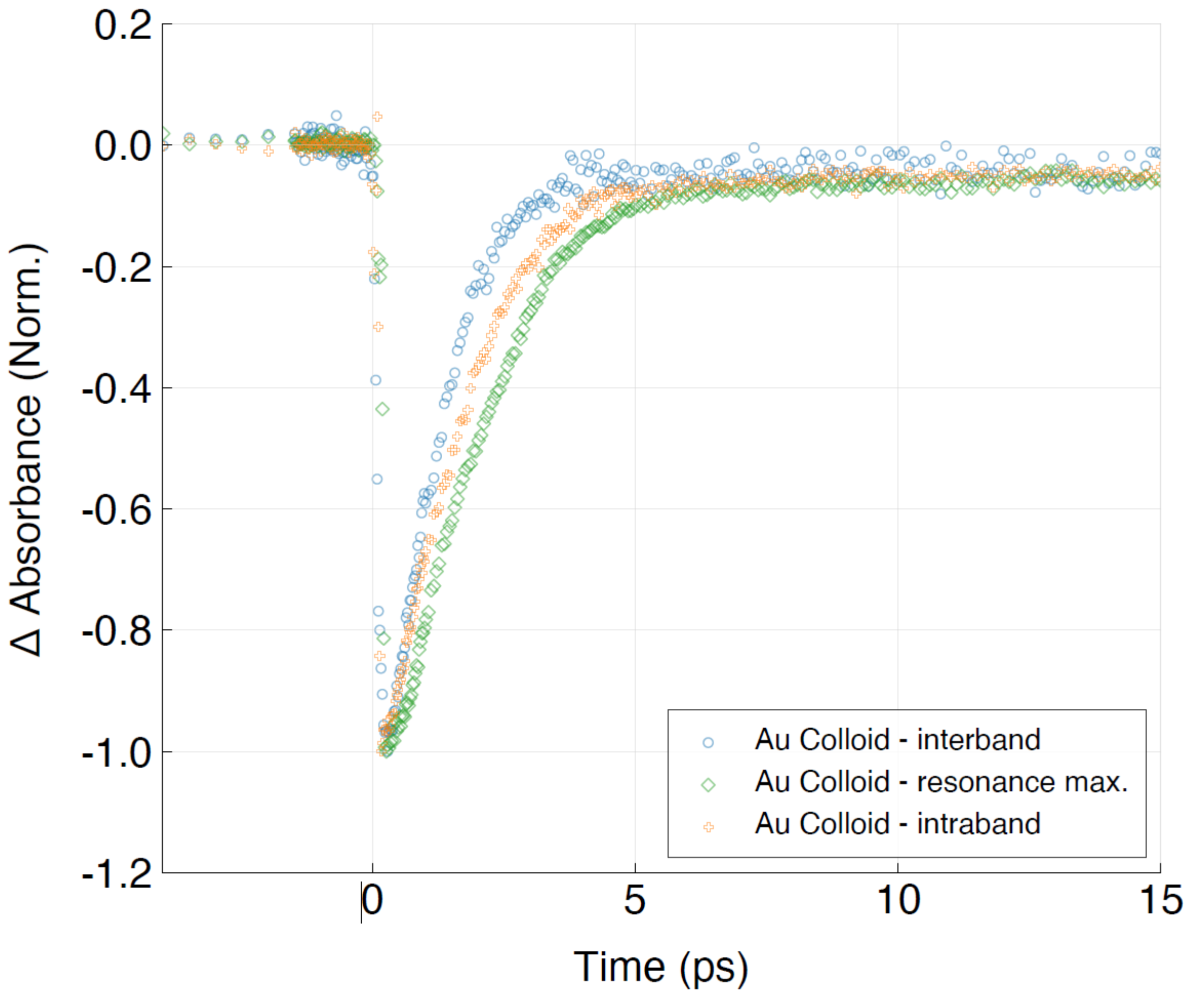

FIGURE S11: Kinetic traces extracted near the excitation wavelength normalized in intensity, showing signal decay after excitations at interband (below the LSPR peak at 450 nm), resonance maximum (at the LSPR peak maximum 520 nm), and intraband (above the LSPR peak at 532 nm).

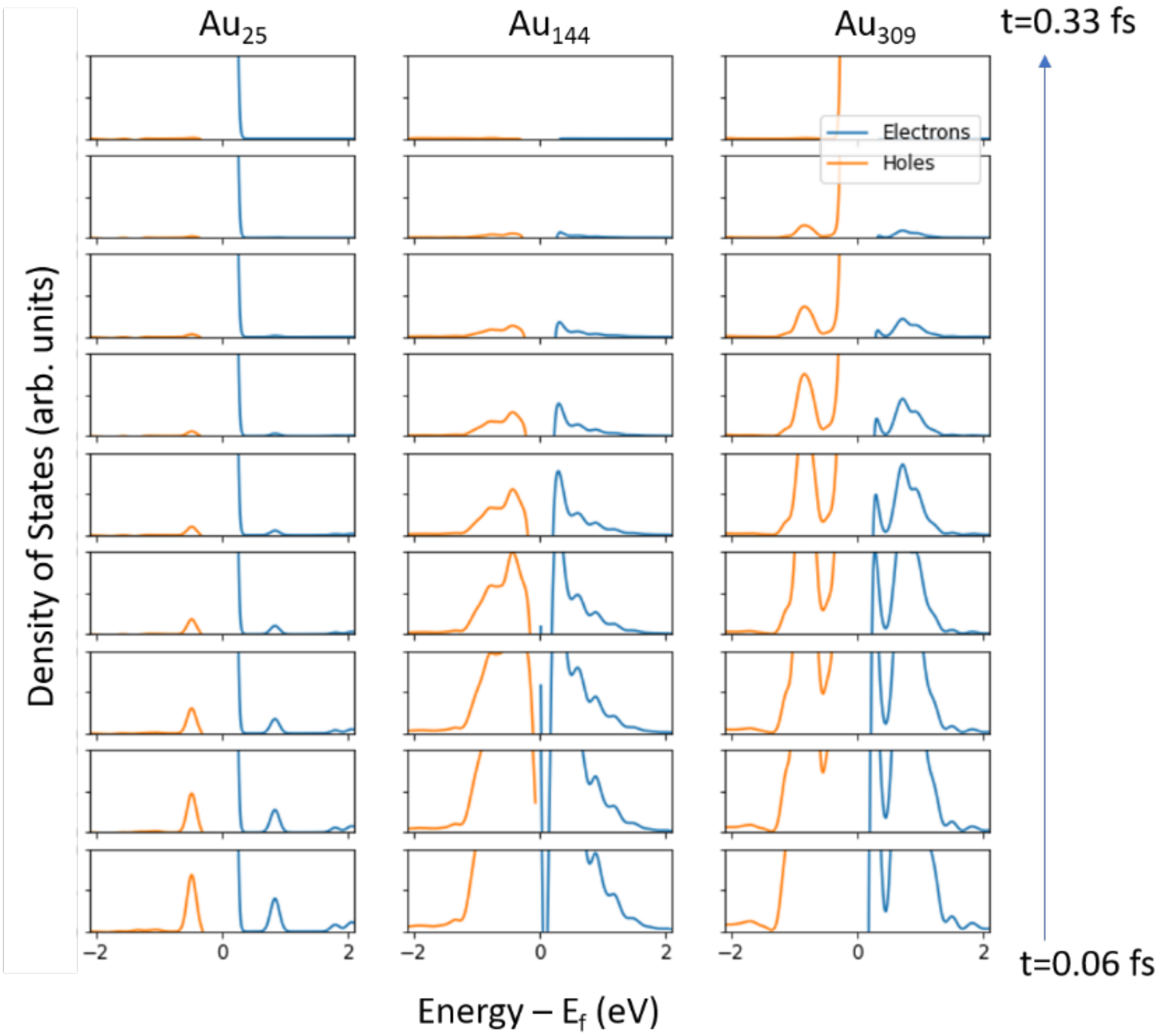

Figure S12: Density of states for hole – electron pairs in excited Au particles simulated using the Ehrenfest ansatz with the DFTB+ code. A 2eV laser pulse energy has been used for the excitation in all cases.

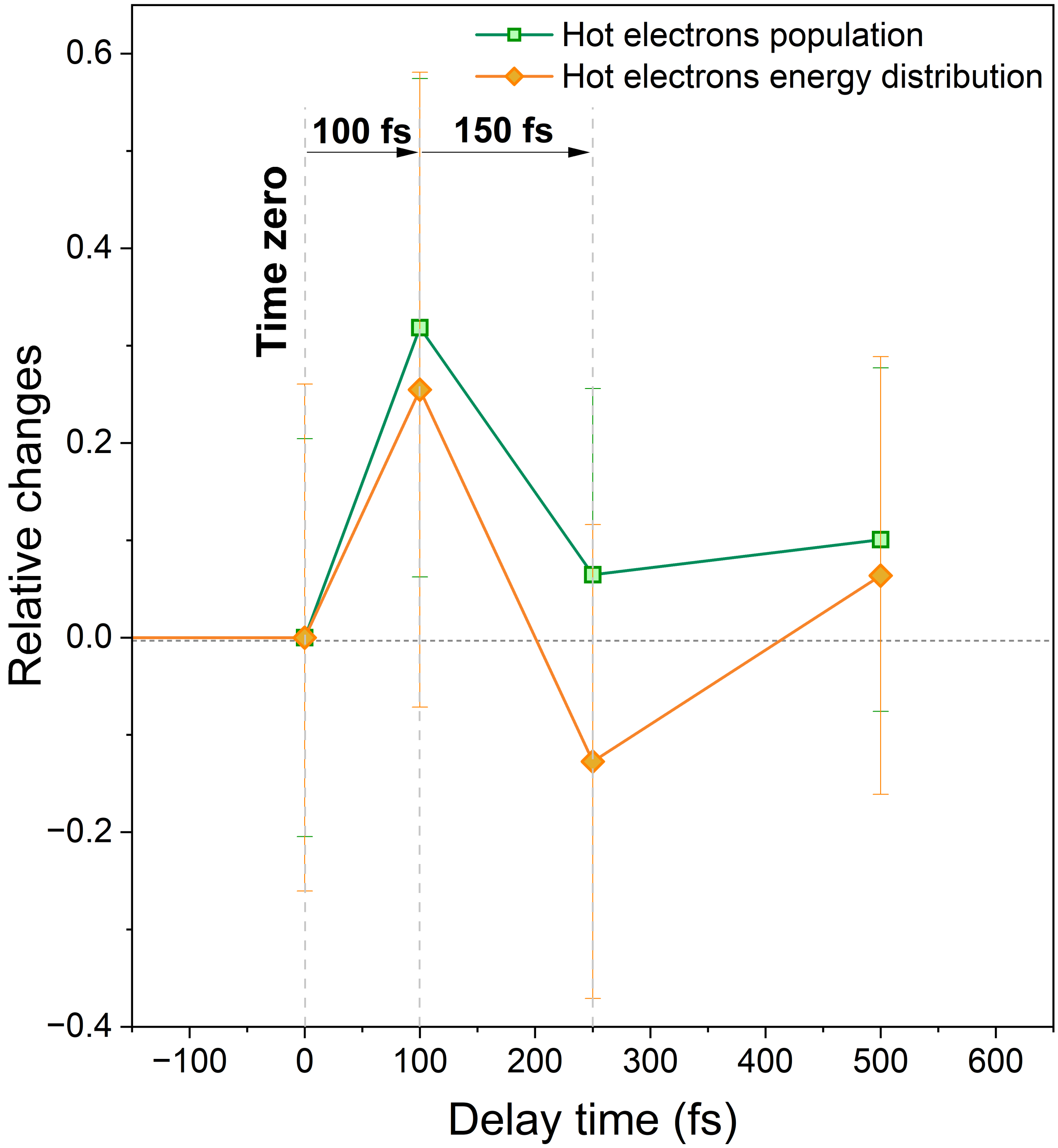

FIGURE S13: Relative changes in hot electrons mean energy distribution (orange trace) and population (green trace).

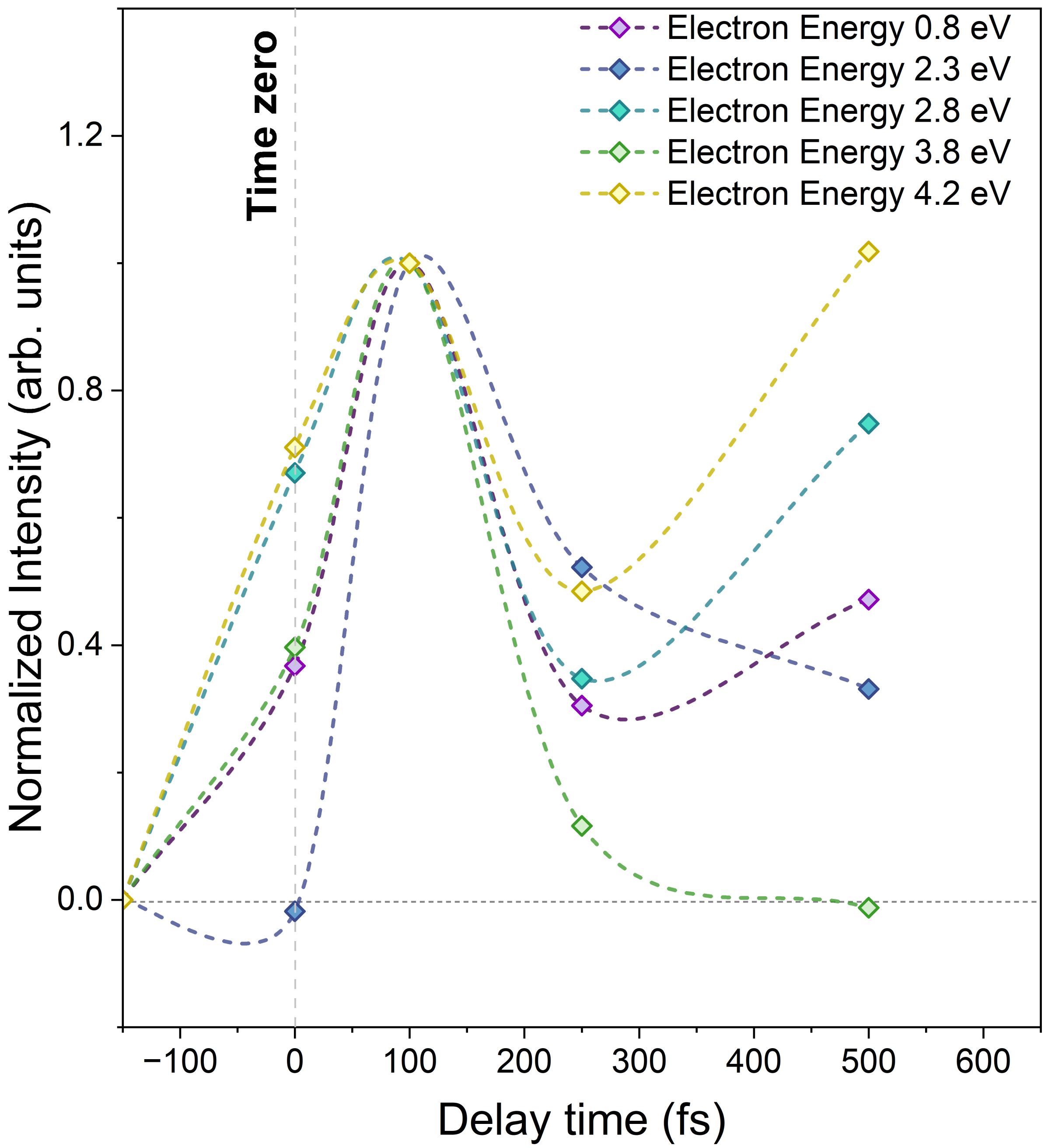

FIGURE S14: Temporal evolution of the hot electrons with different energies indicating that the Fermi-Dirac distribution is not reached within 500 fs. Note the energy of the electrons is affected by the Au core-hole lifetime, while the intensity has a signifcant error associated specially at the longer delay times due to probe lower sensitivity.

Supplementary references:

[1] Piella, J., Bastús, N. G., Puntes, V. Size-Controlled Synthesis of Sub-10-nanometer Citrate-Stabilized Gold Nanoparticles and Related Optical Properties. *Chem. Mater*. **28**, 1066-1075 (2016).

[2] Hattori, Y.; Abdellah, M.; Meng, J.; Zheng, K.; Sá, J. Simultaneous Hot Electron and Hole Injection upon Excitation of Gold Surface Plasmon, *J. Phys. Chem. Lett.* **10**, 3140-3146 (2019).

[3] Szlachetko, J., Szade, J., Beyer, E. et al. SOLARIS national synchrotron radiation centre in Krakow, Poland. *Europ. Phys. J. Plus* **138,** 1-10 (2023).

[4] Ravel, B., Newville, M. ATHENA and ARTEMIS: Interactive graphical data analysis using IFEFFIT. *Phys. Scr*. **2005**, 1007 (2005).

[5] Szczepanik-Ciba, M., Sobol, T., Szade, J. PHELIX–A new soft X-ray spectroscopy beamline at SOLARIS synchrotron. *Nucl. Instrum. Methods Phys. Res., Sect. B* **492**, 49-55 (2021).

[6] Bacellar, C., Kinschel, D., Mancini, G. F., Ingle, R. A., Rouxel, J., Cannelli, O., Cirelli, C., Knopp, G., Szlachetko, J., Lima, F. A. et al. Spin cascade and doming in ferric hemes: Femtosecond X-ray absorption and X-ray emission studies. *Proc. Natl. Acad. Sci. USA* **117**, 21914-21920 (2020).

[7] https://docs.scipy.org/doc/scipy/reference/generated/scipy.optimize.curve_fit.html (Accessed on 2024/02/20)

[8] Zhan, D., Seiler, H., Windsor, Y. W., Ernstorfer, R. Ultrafast lattice dynamics and electron–phonon coupling in platinum extracted with a global fitting approach for time-resolved polycrystalline diffraction data. *Struct. Dyn.* **8**, 064301 (2021).

[9] Durham, D. B., Ophus, C., Siddiqui, K. M., Minor, A. M., Filippetto, D. Accurate quantification of lattice temperature dynamics from ultrafast electron diffraction of single-crystal films using dynamical scattering simulations. *Struct. Dyn.* **9**, 064302 (2022).

[10] Xu, C., Baiz, C. R.Cutting through the Noise: Extracting Dynamics from Ultrafast Spectra Using Dynamic Mode Decomposition. *J. Phys. Chem. A* **127**, 9853-9862 (2023).

[11] https://www.probabilitycourse.com/chapter9/9_1_5_mean_squared_error_MSE.php (Accessed on 2024/02/20)

[12] Elstner, M., Porezag, D., Jungnickel, G., Elsner, J., Haugk, M., Frauenheim, T., Suhai, S., Seifert, G. Self-consistent-charge density-functional tight-binding method for simulations of complex materials properties. *Phys. Rev. B* **58**, 7260 (1998).

[13] Porezag, D., Frauenheim, T., Köhler, T., Seifert, G., Kaschner, R. Construction of tight-binding-like potentials on the basis of density-functional theory: Application to carbon. *Phys. Rev. B* **51**, 12947 (1995).

[14] Aradi, B., Hourahine, B., Frauenheim, T. DFTB+, a Sparse Matrix-Based Implementation of the DFTB Method. *J. Phys. Chem. A* **111**, 5678-5684 (2007).

[15] Fihey, A., Hettich, C., Touzeau, J., Maurel, F., Perrier, A., Köhler, C., Aradi, B., Frauenheim, T. SCC-DFTB parameters for simulating hybrid gold-thiolates compounds. *J. Comp. Chem.* **36**, 2075-2085 (2015).

[16] Bonafé, F. P., Aradi, B., Hourahine, B., Medrano, C. R., Hernández, F. J., Frauenheim, T., Sánchez, C. G. A Real-Time Time-Dependent Density Functional Tight-Binding Implementation for Semiclassical Excited State Electron–Nuclear Dynamics and Pump–Probe Spectroscopy Simulations. J. *Chem. Theory Comp.* **16**, 4454-4469 (2020).
